# Insufficient proximal medullary filling of cephalomedullary nails in intertrochanteric femur fractures predicts excessive postoperative sliding: a case–control study

**DOI:** 10.1186/s12891-023-06213-3

**Published:** 2023-02-28

**Authors:** Shi-Jie Li, Shi-Yi Chen, Shi-Min Chang, Shou-Chao Du, Sun-Jun Hu

**Affiliations:** 1 Department of Orthopaedic Surgery, Yangpu Hospital, School of Medicine, Tongji University, 450 Tengyue Road, Shanghai, 200090 China; 2 Department of Orthopaedic Surgery, Shanghai Fourth People’s Hospital, Tongji University School of Medicine, Shanghai, China

**Keywords:** Intertrochanteric fracture, Medullary filling degree, Excessive sliding, Reduction pattern

## Abstract

**Objective:**

Excessive postoperative sliding is a common complication of intramedullary nails in the treatment of intertrochanteric femur fractures. The aim of this study was to identify risk factors for excessive postoperative sliding in the intertrochanteric fractures treated with an intramedullary nail.

**Methods:**

A retrospective analysis of 369 patients with femoral intertrochanteric fractures treated with short intramedullary nails between February 2017 and September 2020 was performed. Patients were classified into an excessive sliding group (ES group) and a control group according to the sliding distance after 6 months of follow-up. The proximal medullary filling degree (MFD), fracture reduction patterns in the anteroposterior (AP) view and lateral view, and tip-apex distance (TAD) were evaluated and compared in each group.

**Results:**

Thirty-three cases were included in the ES group, and 336 cases were included in the control group. No significant differences in age, sex, fracture side, AO Foundation and Orthopaedic Trauma Association (AO/OTA) classification, Dorr classification, Singh Osteoporosis Index (SOI), American Society of Anesthesiologists classification (ASA), TAD or fracture reduction patterns in the AP view were noted between the two groups. The negative reduction pattern can strongly predict excessive postoperative sliding (OR 4.286, 95% CI 1.637–11.216, *P* = 0.003). The incidence of excessive postoperative sliding increased by 8.713-fold when the MFD decreased by 10% (OR 8.713, 95% CI 1.925–39.437, *P* = 0.005).

**Conclusions:**

A low medullary filling degree and negative fracture reduction pattern in the lateral view were both independent risk factors for excessive postoperative sliding.

**Supplementary Information:**

The online version contains supplementary material available at 10.1186/s12891-023-06213-3.

## Introduction

With the accelerating process of population aging, geriatric hip fracture is becoming a major social health problem worldwide. By 2050, the incidence of hip fracture is expected to be 4.5–6.26 million [1, 2]. Internal fixation remains the preferred treatment for intertrochanteric fractures. However, the failure rate of internal fixation after an intertrochanteric fracture has been reported to be as high as 6%—20% [3], and the functional outcome is not satisfactory. Since the concept of tip-apex distance [4] (TAD) and Cal-TAD [5] has been widely accepted, the incidence of femoral head cut-out has decreased significantly in clinical practice. However, excessive sliding of the helical blade is often observed during follow-up, especially when an intramedullary nail with a sliding helical blade is favored in the clinic.

Many clinicians have recognized the importance of anteromedial cortex buttress and consciously avoid a negative reduction pattern to reduce the incidence of postoperative excessive sliding [6–9]. However, in non-negative reduction patterns, where the anteromedial cortex of the proximal fragment is effectively supported by the anteromedial cortex of the distal fragment, excessive postoperative sliding still occurred. Excessive sliding can lead to hip pain, unacceptable shortening of the femoral neck and other hip dysfunctions [10].

This study aimed to investigate the risk factors of excessive sliding in intertrochanteric fractures. Inadequate medullary filling of cephalomedullary nails may impair the bone-nail overall stability. In this study, we introduce a new method to evaluate the medullary filling degree (MFD) in intertrochanteric fractures treated with cephalomedullary nails. We hypothesize that excessive postoperative sliding was associated with inadequate MFD, reduction patterns, fracture types, TAD, and medullary cavity morphology in intertrochanteric fractures.

## Materials and methods

### Study population

We retrospectively evaluated the data of patients with femoral intertrochanteric fractures who underwent surgery at our hospital from February 2017 to September 2020. Inclusion criteria: 1. Intertrochanteric fractures treated with intramedullary nail with a sliding helical blade (195 mm in length and 10 mm in distal segment diameter); 2. Age ≥ 65. Pathological fractures were excluded from this study. After further excluding 24 patients who lost follow-up, 369 patients were finally enrolled in this study, including 33 patients with excessive sliding (ES group) and 336 patients without excessive sliding (including 3 cut out and 6 varus displacement) (control group). A short curved femoral intertrochanteric nail (FITN) with a sliding helical blade (Beijing BEST Bio-Technical Co. Ltd, Beijing, China) was used for fixation in all cases. This intramedullary nail with anterior curve named BEST-FITN was improved on the basis of PFNA-II according to the proximal femoral morphology of Chinese patients [11]. All BEST-FITN cephalomedullary nails used in this study were 195 mm in length and 10 mm in distal segment diameter.

### Operative technique and perioperative management

Patients were supine on an orthopedic traction table and operated on by the same group of surgeons. Internal fixation was performed after closed reduction, which achieved the Baumgaertner reduction standard and the Chang reduction standard. Weight bearing and isometric quadriceps exercises were allowed and performed on the first day after surgery. Physical therapists were involved in developing rehabilitation protocol for each patient.

### Postoperative evaluation

Patient information, such as age, sex, fracture side, and American Society of Anesthesiologists classification (ASA), was collected from the hospital database. The tip-apex distance (TAD) [4], reduction pattern in AP view and lateral view [12], fracture classification according to the AO/OTA classification, Dorr classification [13], Baumgaertner reduction quality criteria (BRQC) [4], and Singh Osteoporosis Index (SOI) [14] were evaluated on the preoperative or postoperative AP and lateral radiographs. The measurement and classification methods were described in previous studies. The fracture reduction pattern in the AP view and lateral view was distinguished by the classification of Chang [12], and the typical positive, negative and neutral reduction patterns of the radiograph are shown in Figure S1. The AO/OTA classification was used in the 2018 edition without subgroups [15]. Excessive sliding was defined as a lateral blade sliding distance of greater than 10 mm postoperatively, as previously reported. The sliding distance were measured on the 6-months follow-up radiographs and the measurement methods were described by Tsukada [7] in a previous study. All of the parameters were evaluated by two observers. The mean TAD was used, and the assistance of a third observer judged the controversial categorical data.

### Measurement of medullary filling degree in lateral fluoroscopy

**Fig. 1:**
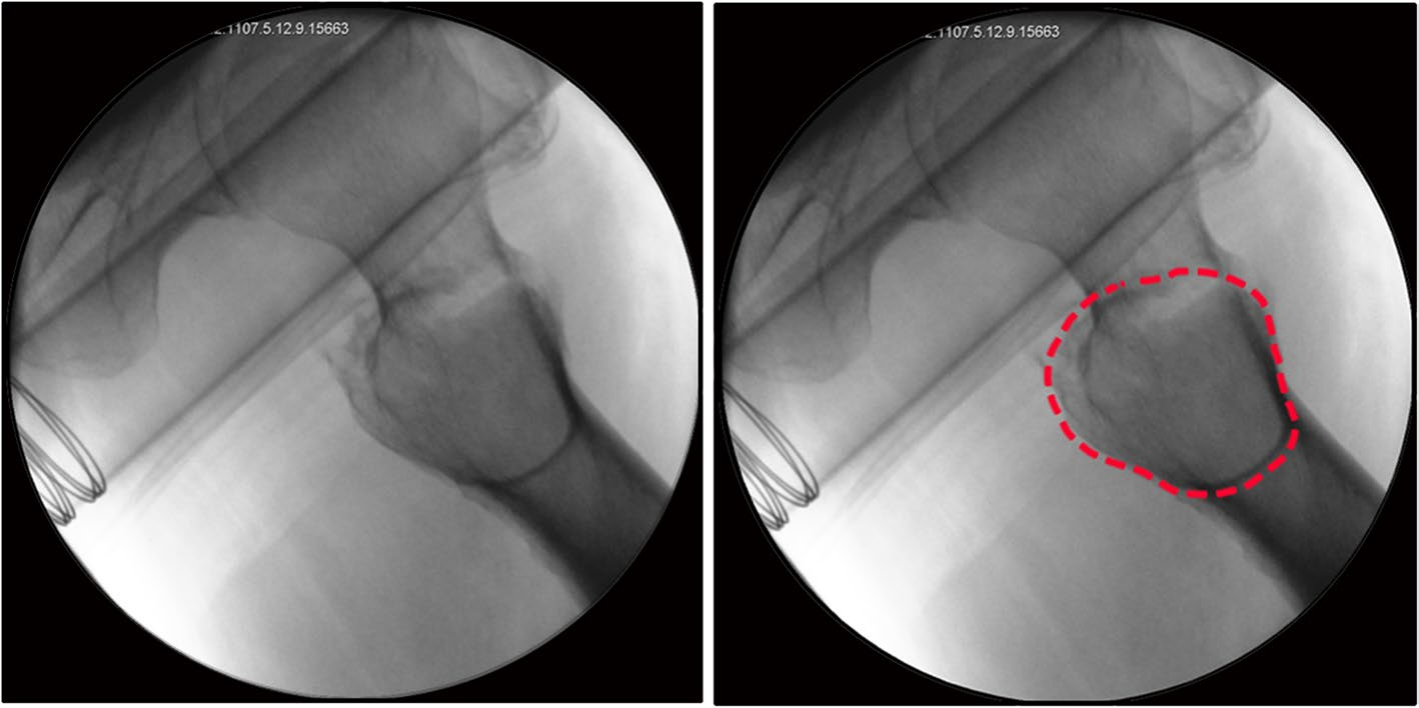
The vacuole sign is shown in the lateral fluoroscopic view (red marked)

Fluoroscopic imaging of the hip on a lateral view is a standard version when the nail, femoral neck, and head are all aligned. In this study, the standard lateral view of the hip was obtained when the direction of the fluoroscope was 45 degrees to the body's long axis on the horizontal plane without abduction or external rotation of the leg (Figure S2). A vacuole sign appeared in the proximal femur on lateral fluoroscopy of the hip, which crossed with the distal medullary cavity and was always clear and recognizable (Fig. 1). We defined the ratio of nail diameter to medullary cavity diameter at the lower edge of the vacuole sign as the medullary filling degree (MFD). The specific measurement methods of MFD are described as follows.

**Fig. 2:**
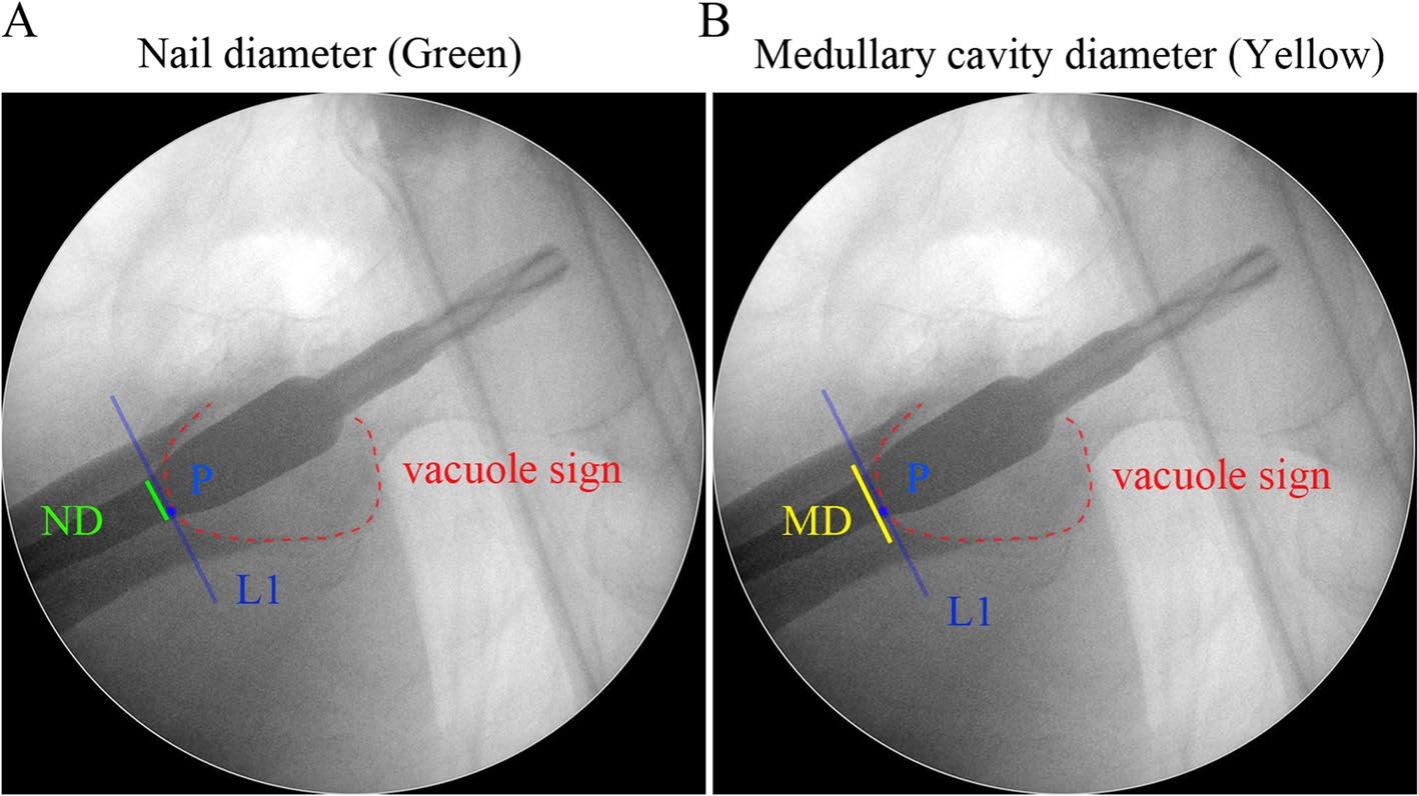
The measurement of MFD. **A**. Measurement of nail diameter ND (green). **B**. Measurement of medullary cavity diameter MD. MFD = ND/MD

We marked the lowest point of the vacuole sign as P on the lateral radiograph. Then, Line L1 was drawn perpendicular to the long axis of the medullary canal through point P. Finally, femoral medullary width FW and intramedullary nail diameter ND were measured on Line L1. The sagittal MFD was calculated as ND/FW (Fig. 2).

### Specimen measurements

Specimen measurements were performed in this study. The inferior border of the vacuole sign shown on the lateral radiograph was actually 15.7 ± 1.26 mm distal to the lower edge of the lesser trochanter on femoral specimens (Table S1). The details of the specimen measurement method are shown in the supplemental files.

### Statistical analyses

Continuous variables were compared using Student’s t test, and categorical variables were compared using the chi-squared test. Odds ratios (ORs) with 95% confidence intervals (CIs) were calculated for dichotomous variables. The significant independent variables from univariate analysis were entered in multivariable binary logistic regression analysis.

Interobserver and intraobserver reliability of TAD and MFD were assessed using the intraclass correlation coefficient (ICC). The interobserver and intraobserver reliability of the SOI, reduction pattern in the AP view, reduction pattern in the lateral view, AO/OTA classification, and Dorr classification were assessed using the kappa coefficient. All statistical analyses were performed using SPSS 22.0 (IBM SPSS Statistics, New York, USA), and significance was set at P less than 0.05.

### Statement

Our work has been reported in line with the STROBE criteria [16].

## Results

**Fig. 3:**
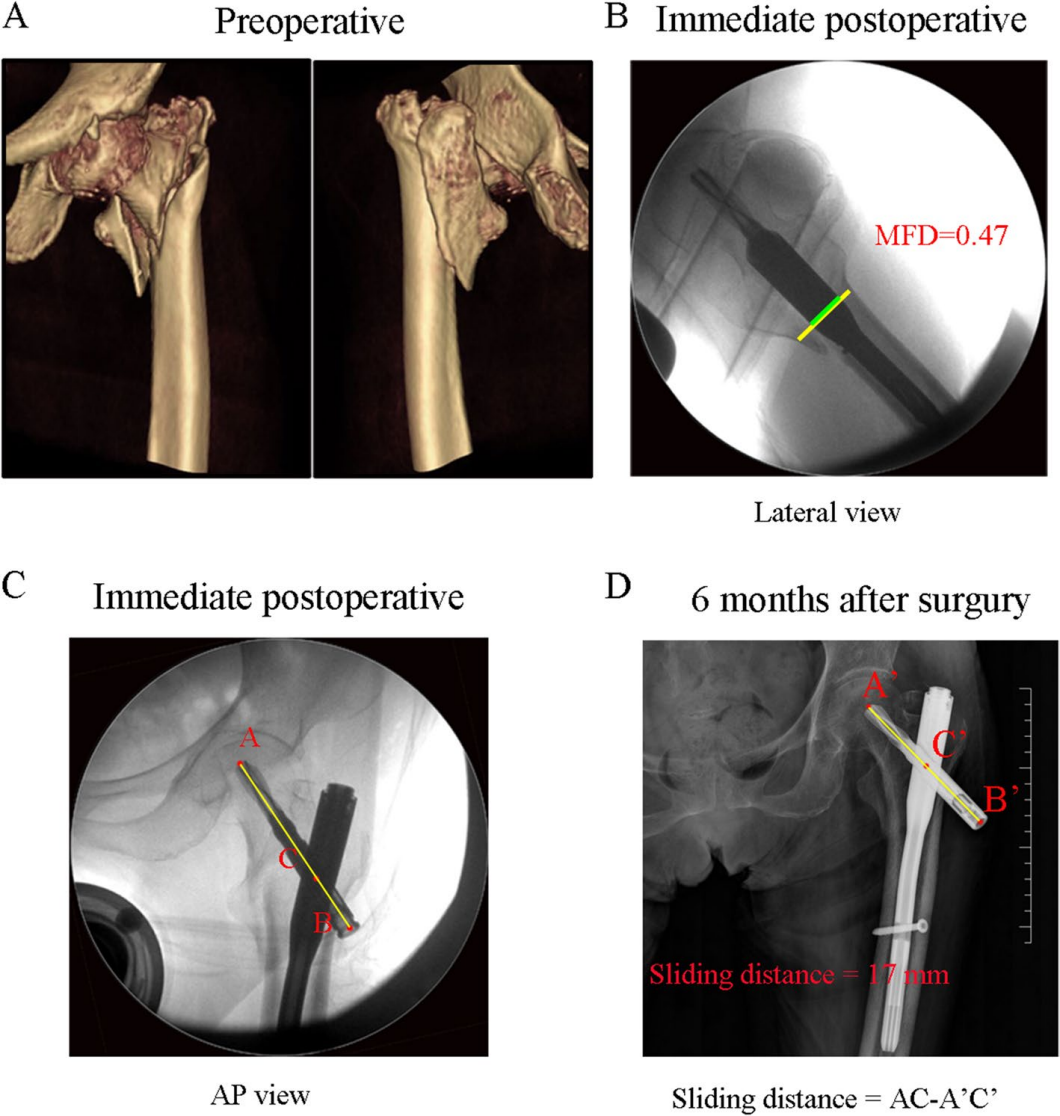
A typical case in the ES group. An 89-year-old women with trochanteric femur fracture by closed reduction and cephalomedullary nail fixation. **A**. The fracture type was classified as 2018 AO/OTA 31 A2. **B**-**C** Immediate postoperative fluoroscopy in lateral and AP view showed a good reduction quality and the MFD was 47%. **D**. Follow-up X-ray in 6 months. The AP view showed over-sliding of the helical blade. The sliding distance was 17 mm

**Fig. 4:**
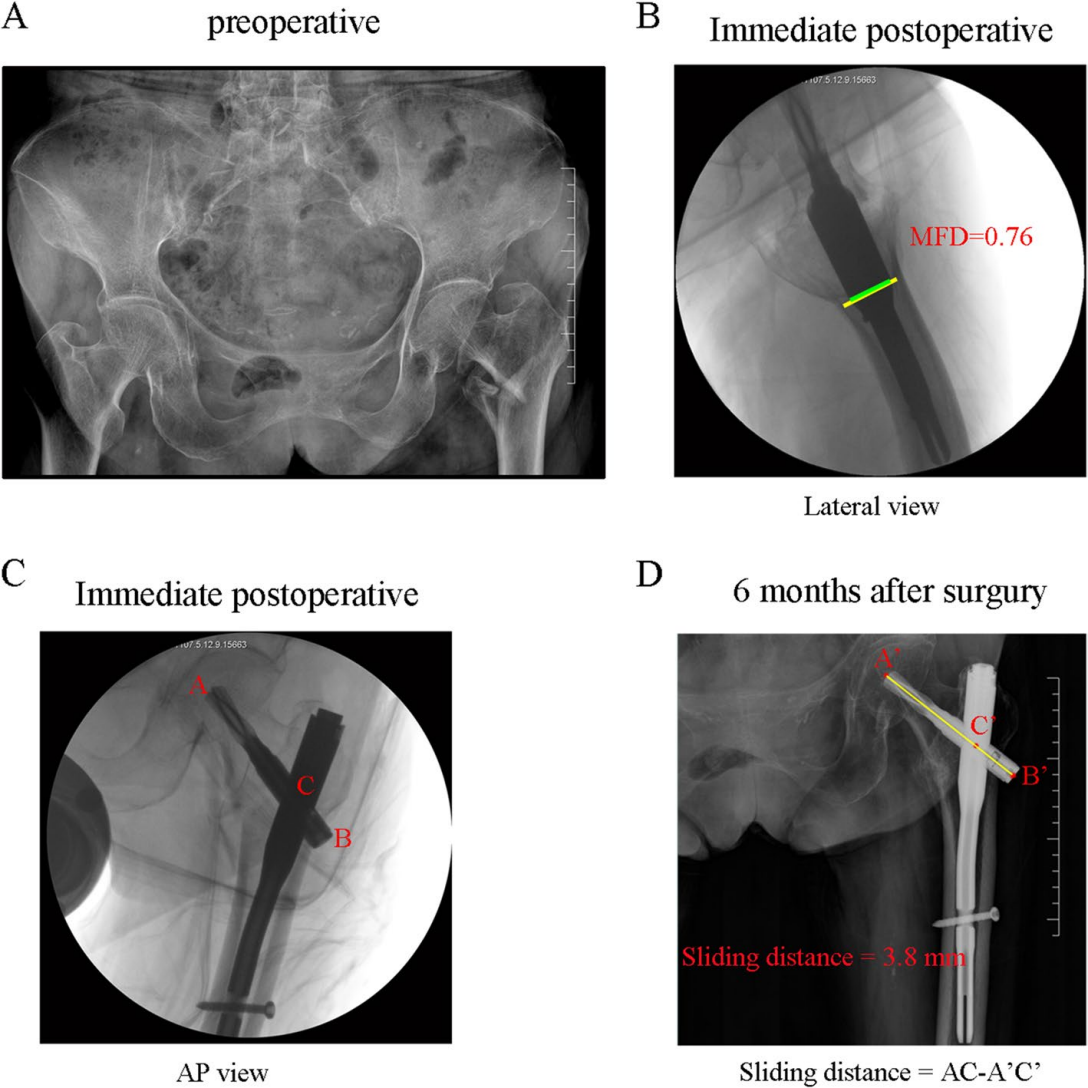
A typical case in the control group. An 89-year-old women with trochanteric femur fracture by closed reduction and cephalomedullary nail fixation. **A**. The fracture type was classified as 2018 AO/OTA 31 A2. **B**-**C** Immediate postoperative fluoroscopy in lateral and AP view showed a good reduction quality and the MFD was 76%. **D**. Follow-up X-ray in 6 months. The AP view showed slight backout of the helical blade. The sliding distance was 3.8 mm

There were 369 cases of intertrochanteric fractures included in this study. Intramedullary nails 195 mm long were used to fix all patients. Thirty-three of the 369 fractures exhibited excessive sliding and were included in the excessive sliding group (ES group). The typical case is shown in Fig. 3. The other 336 fractures were included in the control group. The typical case is shown in Fig. 4.

### Univariate analysis

No significant difference in age, sex, fracture side, AO/OTA classification, Dorr classification, SOI, or ASA were noted between the ES and control groups (Table 1). The mean TAD in the ES group was 19.81 ± 2.48 compared with 18.86 ± 3.54 in the control group, and the difference between the two groups was not significant. In the reduction quality analysis, patients in the ES group exhibited a significantly different reduction pattern in the lateral view compared with the control group. However, BRQC and fracture reduction pattern in AP view did not significantly differ between the two groups. The MFD was significantly lower in the ES group than in the control group.

**Table 1:** Univariate analysis of patient demographics and clinical characteristics

Characteristic	ES group	Control group	*P*-value
age	80.91 ± 8.58	82.50 ± 7.12	0.491^&^
Gender (Male/Female)	33(6/27)	336(99/237)	0.429 ^‡^
side (Left/Right)	33(18/15)	336(153/183)	0.567^‡^
TAD	19.81 ± 2.48	18.86 ± 3.54	0.389 ^&^
MFD	0.59 ± 0.06	0.71 ± 0.09	0.000^&*^
**AO/OTA classification**			0.380^‡^
A1	0	42	
A2	33	285	
A3	0	9	
**BRQC**			0.541^‡^
good	3	54	
acceptable	30	282	
**Reduction pattern in AP view**			0.831^‡^
Positive	6	78	
Neutral	27	252	
Negative	0	6	
**Reduction pattern in Lateral view**			0.000^‡*^
Positive	0	12	
Neutral	12	288	
Negative	21	36	
**SOI**			0.834^‡^
1	0	6	
2	6	69	
3	18	117	
4	9	129	
5	0	12	
6	0	3	
**Dorr classification**			0.612^‡^
A	3	69	
B	21	201	
C	9	66	
**ASA**			0.891^‡^
I	3	24	
II	12	159	
III	18	150	
IV	0	3	
**Follow-up (month)**	15.82 ± 7.05	13.62 ± 4.55	0.591^&^

^&^Student’s t test

^‡^Chi-squared test

^*^Statistically significant

In conclusion, both MFD and the reduction pattern in the lateral view may be associated with excessive postoperative sliding. However, none of the other parameters showed significant differences between the two groups. The interobserver agreement and intraobserver agreement are shown in Table 2. The reliability coefficient was good for all parameters except SOI and Dorr classification.

**Table 2:** Intraclass correlation coefficient of paraments measured in research

Paraments	Interobserver agreement (ICC or κ, 95% CI)	Intraobserver agreement (Icc or κ, 95% CI)
MFD, ICC	0.845 (0.785–0.889)	0.825 (0.759–0.874)
Tip-apex distance, ICC	0.936 (0.910–0.955)	0.887 (0.843–0.920)
SOI, κ	0.462 (0.348–0.576)	0.390 (0.280–0.499)
Reduction pattern in AP view, κ	0.914 (0.829–0.998)	0.893 (0.801–0.985)
Reduction pattern in Lateral view, κ	0.878 (0.774–0.982)	0.873 (0.765–0.981)
AO/OTA classification, κ	0.721 (0.549–0.893)	0.619 (0.421–0.817)
Dorr classification, κ	0.755 (0.651–0.859)	0.587 (0.471–0.703)

*ICC* Intraclass correlation coefficient, *κ* Kappa coefficient, *CI* Confidence interval

### Negative reduction pattern on the lateral view is an independent risk factor for excessive postoperative sliding

In the ES group, there were 12 patients with neutral patterns and 21 with negative patterns on the lateral view. In the control group, there were 12 patients with positive patterns, 288 with neutral patterns, and 36 with negative patterns in the lateral view (Table 1). We further divide the reduction mode into negative and nonnegative (including positive and neutral). A negative reduction pattern can strongly predict excessive postoperative sliding (OR 14.583, 95% CI 3.718–57.198) (Table 3). On the lateral view, the incidence of excessive sliding was increased 14.583-fold in negative patterns compared with nonnegative patterns. After adjustment by multivariate binary logistic regression analysis, the incidence of excessive sliding was increased 4.286-fold in negative patterns compared with nonnegative patterns in the lateral view (OR 4.286, 95% CI 1.637–11.216, *P* = 0.003) (Table 4).

**Table 3:** The results of univariate binary logistic regression analysis

Characteristic	ES group	Control group	OR (95% CI)	*p*-value
**Reduction pattern in Lateral view**			14.583 (3.718–57.198)	0.000^*^
Non-negative	12	300		
Negative	21	36		
**MFD**			28.621 (3.510–233.407)	0.000^*^
> 65%	3	249		
≤ 65%	30	87		
**MFD**			6.944 (1.838–26.239)	0.001^*^
> 60%	18	300		
≤ 60%	15	36		

^*^Statistically significant

**Table 4:** The results of multivariate binary logistic regression analysis

Characteristic	Exp(B) (95% CI)	*p*-value
Reduction pattern in Lateral view	4.286 (1.637–11.216) ^†^	0.003^*^
MFD	8.713 (1.925–39.437) ^#^	0.005^*^

^†^This is a comparison between negative reduction patterns and nonnegative reduction patterns

^#^The odds ratio was calculated by considering a 10% increase in MFD

^*^Statistically significant

### Low MFD is another independent risk factor for excessive postoperative sliding

**Fig. 5:**
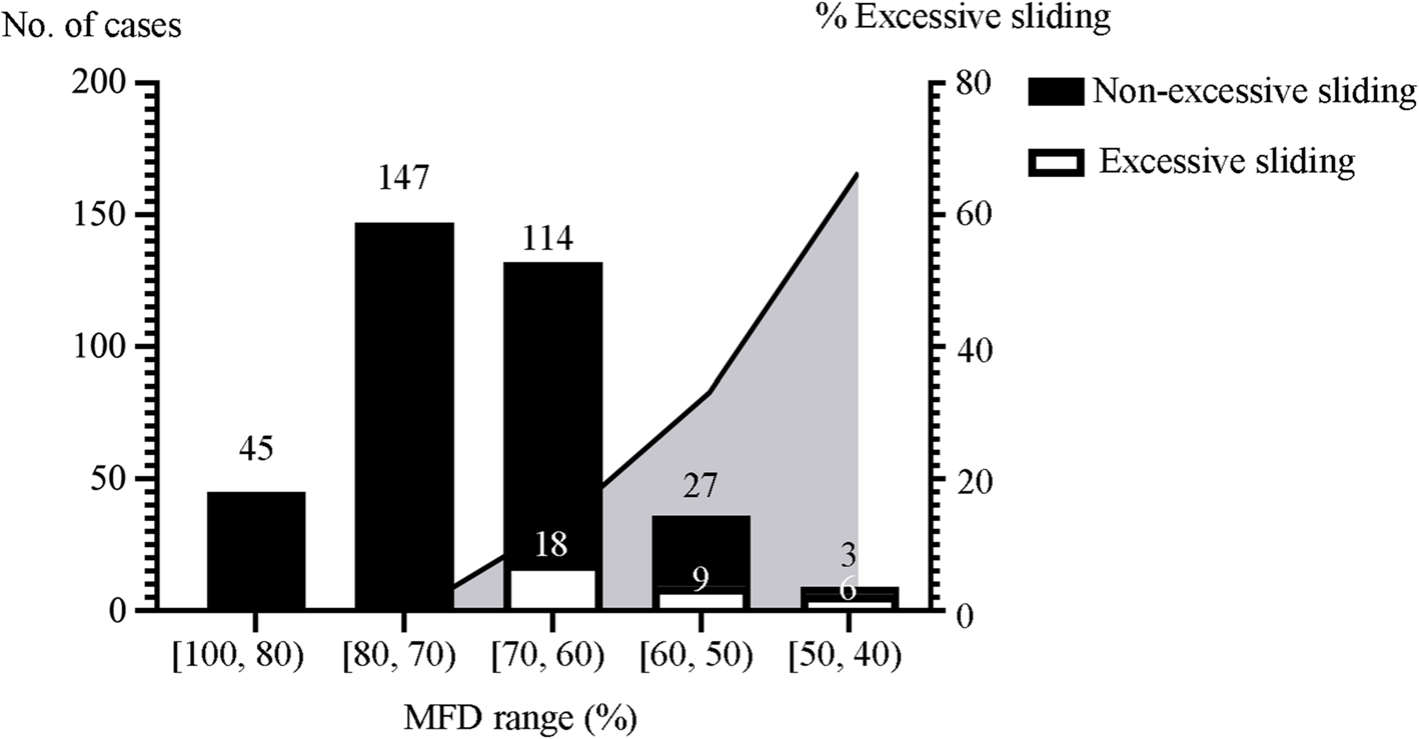
The distribution and proportion of excessive sliding in different ranges of MFDs

In terms of proximal medullary filling degree in lateral view, the mean MFD of the ES group was 59% ± 6%, whereas that of the control group was 71% ± 9%. The difference between the two groups was significant. Most of the MFD values were in the range of 60% to 80%. None of the 369 patients with an MFD of greater than 70% exhibited excessive postoperative sliding. Although one patient with an MFD of 68% exhibited excessive sliding, the rate of excessive sliding for the 252 patients who had an MFD of 65% or greater was only 1.2% (three) compared with 25.6% (thirty) for the 117 patients with an MFD of less than 65%. Compared with MFD > 65%, the OR of MFD ≤ 65% was 28.621 (95% CI 3.510–233.407, *P* = 0.000). Of the 324 patients with MFD > 60%, 18 (5.6%) experienced excessive sliding compared with 15 of 45 patients with an MFD < 60% (Fig. 5). Compared with MFD > 60%, the OR of MFD ≤ 60% was 6.944 (95% CI 1.838–26.239, *P* = 0.001). Furthermore, we included positive results in the univariate analysis in the multivariate binary logistic regression analysis. A direct relationship was demonstrated between a decrease in MFD and an increased risk of excessive postoperative sliding. The incidence of excessive postoperative sliding increased by 8.713-fold when MFD decreased by 10% (OR 8.713, 95% CI 1.925–39.437, *P* = 0.005) (Table 4).

## Discussion

Internal fixation with a sliding device has been used in the treatment of intertrochanteric fractures since the 1970s. The sliding system allows proximal fragment telescopes along the helical blade after loading, which increases osseous contact between the proximal and distal fragment, maintains a constant neck-shaft angle, and reduces the risk of cut-out, cut through, and nonunion compared with previous implants without a sliding mechanism [17, 18]. However, this sliding system may also increase the risk of excessive backout of lag screw/ helical blade [19, 20], resulting in hip pain, unacceptable shortening, or rotation deformity of the limb with poor function [21, 22].

In intertrochanteric fractures, most bone-to-bone impaction occurs on the posterior cortex, and the posterior cortex is thin and often comminuted when the fracture occurs [7, 23]. In contrast, the anteromedial cortex is thick and reliably provides support, limits sliding of the head-neck fragment, maintains the length of the femoral neck, and reduces the sliding distance of the helical blade [12, 24]. Rha et al. retrospectively analyzed 76 intertrochanteric fractures fixed using 135° sliding compression screws and plates and found that excessive postoperative sliding was associated with the loss of the buttress of the anterior or medial cortex [25]. In the current clinical consensus, the negative reduction pattern in the AP view before nail insertion was inadequate reduction and needed further manipulation. In addition, it is easy to transform a negative medial cortical position to a positive or neutral relation in the AP view by manual fracture reduction. This enables the negative reduction pattern in the AP view to rarely occur in reality and makes the comparison of different reduction patterns in the AP view with excessive sliding less relevant [26]. Therefore, many researchers highlight the importance of reduction in the lateral view and indicate that excessive sliding is significantly correlated with negative reduction patterns on the sagittal plane in the lateral view [6–9, 26]. In this study, our results also correspond with this point.

A negative reduction pattern in the lateral view indicates a loss of the buttressing effect of the anterior cortex and instability of bone-nail construction on the sagittal plane. After loading, the proximal fragment together with the intramedullary nail tends to swing sagittally with the distal locking screw as the axis. The anterior cortical nonnegative buttress pattern we have advocated can effectively resist this trend of sagittal movement through the bone to bone buttress on the anterior cortex and enhance the stability of the bone-nail construction on the sagittal plane.

Previous studies suggested that the degree of nail movement in the intramedullary region is inversely correlated with nail diameter and directly correlated with the instability of fracture [27]. In this view, large-diameter intramedullary nails and small anteroposterior-diameter medullary cavities are also beneficial for the stability of the bone-nail constructure. We expect the ratio of the intramedullary nail diameter to medullary cavity diameter to reflect the stability of the bone-nail structure in the sagittal plane. The posterior cortex of the proximal femur is often comminuted in intertrochanteric fractures (A2 type) and is unable to limit the sagittal movement of nails. Therefore, it is reasonable to evaluate the degree of nail movement on the sagittal plane at the fracture line's distal level. According to the study of intertrochanteric fracture maps, the mean distal cortical extension of the fracture line from the lower edge of the lesser trochanter was 13 mm [28, 29]. The low edge of the vacuole sign that appeared on the lateral view of fluoroscopy was 15.7 ± 1.26 mm to the lower edge of the lesser trochanter on the femoral specimens. Thus, it is reasonable to assess the degree of nail movement on the sagittal plane based on the ratio of nail diameter to medullary cavity diameter at the lower edge of the vacuole sign.

Previous studies were too complex due to the measurement method of the filling degree of the medullary cavity. The clinical guidance and application were weak [27]. Some studies only took the diameter of the intramedullary nail into account without regard to the medullary cavity diameter [30]. Compared with previous methods, our measurement of MFD is more reasonable and easier to perform. Importantly, the MFD was measured by intraoperative fluoroscopic images in this study. Therefore, MFD can be easily estimated during an operation to provide a valid reference for intraoperative intramedullary nail selection and placement.

Our study suggested that MFD should be greater than 60%, preferably greater than 65%. This requirement indicates to choose an intramedullary nail with a larger diameter as much as possible, provided that it can be manually inserted without shaft reaming in geriatrics. Moderately increasing the intramedullary nail diameter can reduce the sway range of intramedullary nails in the medullary cavity and improve the overall stability of bone-nail construction in the sagittal plane. Current intramedullary nails are primarily large diameter in the proximal segment and small diameter in the distal segment. Therefore, it is also beneficial to place intramedullary nails as deep as possible. The moderate increase in the sagittal diameter of the nail may also represent an improved approach for intramedullary nailing. We believe that sagital MFD in lateral view is helpful in predicting excessive postoperative sliding. The routine intraoperative estimation of MFD can increase the surgeon’s awareness of the incidence of excessive sliding and help guide operative decision-making.

Despite these findings, this study had limitations. As a single-center retrospective study, it has limitations in generalizability and selection bias, which limits its applicability. All of the CT data used in this study were obtained from Asians. Whether the results would be suitable for other ethnicities requires further investigation.

## Conclusion

In conclusion, a low medullary filling degree and negative fracture reduction pattern in the lateral view were both independent risk factors for excessive postoperative sliding.

## Supplementary Information

**Additional file 1:** **Figure S1.** Schematicillustration of positive, neutral and negative reduction patterns. **Figure S2.** Thedirection of the fluoroscope when obtaining the lateral view of the hip. **Figure S3.** Steel wirewas employed to mark femoral specimen. **Table S1.** The actual position of the inferiorborder of the vacuole sign on the femoral specimens. 

## Data Availability

The dataset used and/or analysed during the current study are available from the corresponding author on reasonable request.

## Abbreviations

ES group: Excessive sliding group
MFD: Medullary filling degree
AP: Anteroposterior
TAD: Tip-apex distance
AO/OTA: AO Foundation and Orthopaedic Trauma Association
SOI: Singh Osteoporosis Index
ASA: American Society of Anesthesiologists classification
BRQC: Baumgaertner reduction quality criteria
ORs: Odds ratios
CIs: Confidence intervals
ICC: Intraclass correlation coefficient
FITN: Femoral intertrochanteric nail

## References

[1] Panteli M, Rodham P, Giannoudis PV (2015). Biomechanical rationale for implant choices in femoral neck fracture fixation in the non-elderly. Injury.

[2] Gullberg B, Johnell O, Kanis JA (1997). World-wide projections for hip fracture. Osteoporos Int.

[3] Chehade MJ, Carbone T, Awwad D, Taylor A, Wildenauer C, Ramasamy B (2015). Influence of Fracture Stability on Early Patient Mortality and Reoperation After Pertrochanteric and Intertrochanteric Hip Fractures. J Orthop Trauma.

[4] Baumgaertner MR, Curtin SL, Lindskog DM, Keggi JM (1995). The value of the tip-apex distance in predicting failure of fixation of peritrochanteric fractures of the hip. J Bone Joint Surg Am.

[5] Kuzyk PR, Zdero R, Shah S, Olsen M, Waddell JP, Schemitsch EH (2012). Femoral head lag screw position for cephalomedullary nails: a biomechanical analysis. J Orthop Trauma.

[6] Kozono N, Ikemura S, Yamashita A, Harada T, Watanabe T, Shirasawa K (2014). Direct reduction may need to be considered to avoid postoperative subtype P in patients with an unstable trochanteric fracture: a retrospective study using a multivariate analysis. Arch Orthop Trauma Surg.

[7] Tsukada S, Okumura G, Matsueda M (2012). Postoperative stability on lateral radiographs in the surgical treatment of pertrochanteric hip fractures. Arch Orthop Trauma Surg.

[8] Tufescu T, Sharkey B (2013). The lateral radiograph is useful in predicting shortening in 31A2 pertrochanteric hip fractures. Can J Surg.

[9] Takigawa N, Moriuchi H, Abe M, Yasui K, Eshiro H, Kinoshita M (2014). Complications and fixation techniques of trochanteric fractures with the TARGON((R)) PF. Injury.

[10] Goto K, Murakami T, Saku I (2022). Postoperative subtype P as a risk factor for excessive postoperative sliding of cephalomedullary nail in femoral trochanteric fractures in old patients: A case series of 263 patients using computed tomography analysis. Injury.

[11] Chang SM, Hu SJ, Ma Z, Du SC, Zhang YQ (2018). Femoral intertrochanteric nail (fitn): a new short version design with an anterior curvature and a geometric match study using post-operative radiographs. Injury.

[12] Chang SM, Zhang YQ, Ma Z, Li Q, Dargel J, Eysel P (2015). Fracture reduction with positive medial cortical support: a key element in stability reconstruction for the unstable pertrochanteric hip fractures. Arch Orthop Trauma Surg.

[13] Dorr LD, Faugere MC, Mackel AM, Gruen TA, Bognar B, Malluche HH (1993). Structural and cellular assessment of bone quality of proximal femur. Bone.

[14] Singh M, Riggs BL, Beabout JW, Jowsey J. Femoral trabecular pattern index for evaluation of spinal osteoporosis. A detailed methodologic description. Mayo Clin Proc. 1973;48:184–9. 4690324

[15] Meinberg EG, Agel J, Roberts CS, Karam MD, Kellam JF (2018). Fracture and Dislocation Classification Compendium-2018. J Orthop Trauma.

[16] Vandenbroucke JP, von Elm E, Altman DG, Gotzsche PC, Mulrow CD, Pocock SJ (2007). Strengthening the Reporting of Observational Studies in Epidemiology (STROBE): explanation and elaboration. PLoS Med.

[17] Lenich A, Vester H, Nerlich M, Mayr E, Stockle U, Fuchtmeier B (2010). Clinical comparison of the second and third generation of intramedullary devices for trochanteric fractures of the hip–Blade vs screw. Injury.

[18] Verettas DA, Ifantidis P, Chatzipapas CN, Drosos GI, Xarchas KC, Chloropoulou P (2010). Systematic effects of surgical treatment of hip fractures: gliding screw-plating vs intramedullary nailing. Injury.

[19] Yoo JH, Kim TY, Chang JD, Kwak YH, Kwon YS (2014). Factors influencing functional outcomes in united intertrochanteric hip fractures: a negative effect of lag screw sliding. Orthopedics.

[20] Maniscalco P, Bertone C, Rivera F, Urgelli S (2002). Use of a modified IMHS for unstable intertrochanteric fractures. J Orthop Traumatol.

[21] Baixauli F, Vicent V, Baixauli E, Serra V, Sanchez-Alepuz E, Gomez V, et al. A reinforced rigid fixation device for unstable intertrochanteric fractures. Clin Orthop Relat Res. 1999:205–15. 10.1097/00003086-199904000-00027 10212615

[22] Chan KC, Gill GS. Cemented hemiarthroplasties for elderly patients with intertrochanteric fractures. Clin Orthop Relat Res. 2000:206–15. 10.1097/00003086-200002000-00025 10693568

[23] Carr JB (2007). The anterior and medial reduction of intertrochanteric fractures: a simple method to obtain a stable reduction. J Orthop Trauma.

[24] Chang SM, Hou ZY, Hu SJ, Du SC (2020). Intertrochanteric Femur Fracture Treatment in Asia: What We Know and What the World Can Learn. Orthop Clin North Am.

[25] Rha JD, Kim YH, Yoon SI, Park TS, Lee MH (1993). Factors affecting sliding of the lag screw in intertrochanteric fractures. Int Orthop.

[26] Ito J, Takakubo Y, Sasaki K, Sasaki J, Owashi K, Takagi M (2015). Prevention of excessive postoperative sliding of the short femoral nail in femoral trochanteric fractures. Arch Orthop Trauma Surg.

[27] Durusoy S, Paksoy AE, Korkmaz M, Daglar B, Elibol FKE (2021). The effect of medullary fill on varus collapse in AO 31A3 intertrochanteric (reverse obliquity) fracture treated with cephalomedullary nails. Orthop Traumatol Surg Res.

[28] Xiong WF, Zhang YQ, Chang SM, Hu SJ, Du SC (2019). Lesser Trochanteric Fragments in Unstable Pertrochanteric Hip Fractures: A Morphological Study Using Three-Dimensional Computed Tomography (3-D CT) Reconstruction. Med Sci Monit.

[29] Fu Y, Liu R, Liu Y, Lu J (2019). Intertrochanteric fracture visualization and analysis using a map projection technique. Med Biol Eng Comput.

[30] Cheung ZB, Selverian S, Barbera J, Forsh DA (2020). The effect of nail diameter on proximal femoral shortening after internal fixation of pertrochanteric hip fractures with short cephalomedullary nails. J Orthop.

