# Supplementary material for: Insufficient proximal medullary filling of cephalomedullary nails in intertrochanteric femur fractures predicts excessive postoperative sliding: a case–control study
Source: BMC Musculoskelet Disord. 2023 Feb 28;24:156. doi: 10.1186/s12891-023-06213-3 (PMC9972673; doi:10.1186/s12891-023-06213-3)
Supplement: Supplementary file 1 — Additional file 1: Figure S1. Schematicillustration of positive, neutral and negative reduction patterns. Figure S2. Thedirection of the fluoroscope when obtaining the lateral view of the hip. Figure S3. Steel wirewas employed to mark femoral specimen. Table S1. The actual position of the inferiorborder of the vacuole sign on the femoral specimens. [file 12891_2023_6213_MOESM1_ESM.docx]

**Supplemental**

**Supplemental Material and Method**

**Specimens measurement**

Ten femur specimens were enrolled in this study. Steel wires were employed to mark femoral specimens, and the direction of the fluoroscopic was 45 degrees to the long axis of femoral specimens on the horizontal plane. Femur specimens were marked with steel wires over the medial cortex separately, and a typical graph is shown in Figure S3A and Figure S3B. After obtaining standard femur neck lateral views under C-arm fluoroscopy, we moved the wire position along the medial cortex toward the distal femur until the wire was at the lower edge of the vacuole sign in the lateral view (Figure S3C). Then, the distance from the position of the wire to the lower edge of the lesser trochanter was recorded.

**Figure S1**

**
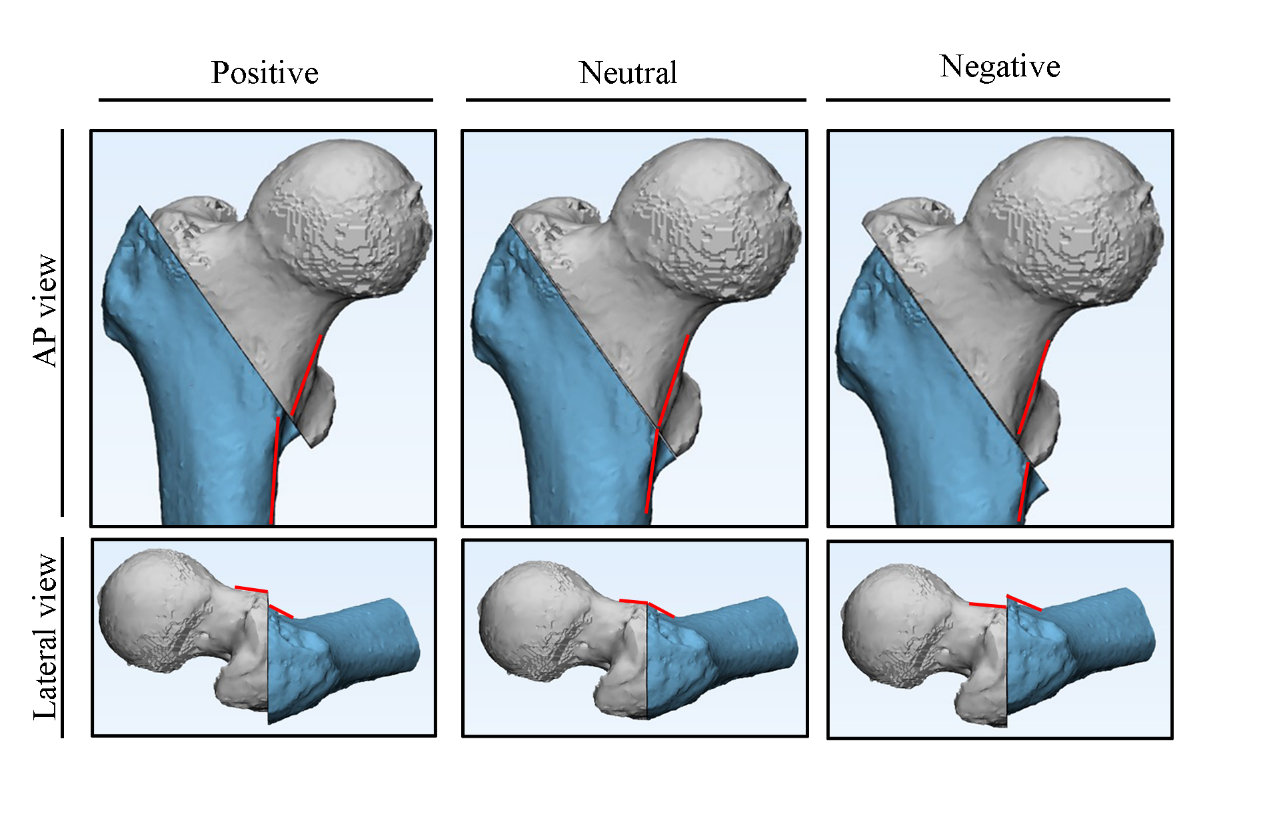
**

Figure S1. Schematic illustration of positive, neutral and negative reduction patterns.

**Figure S2**

**
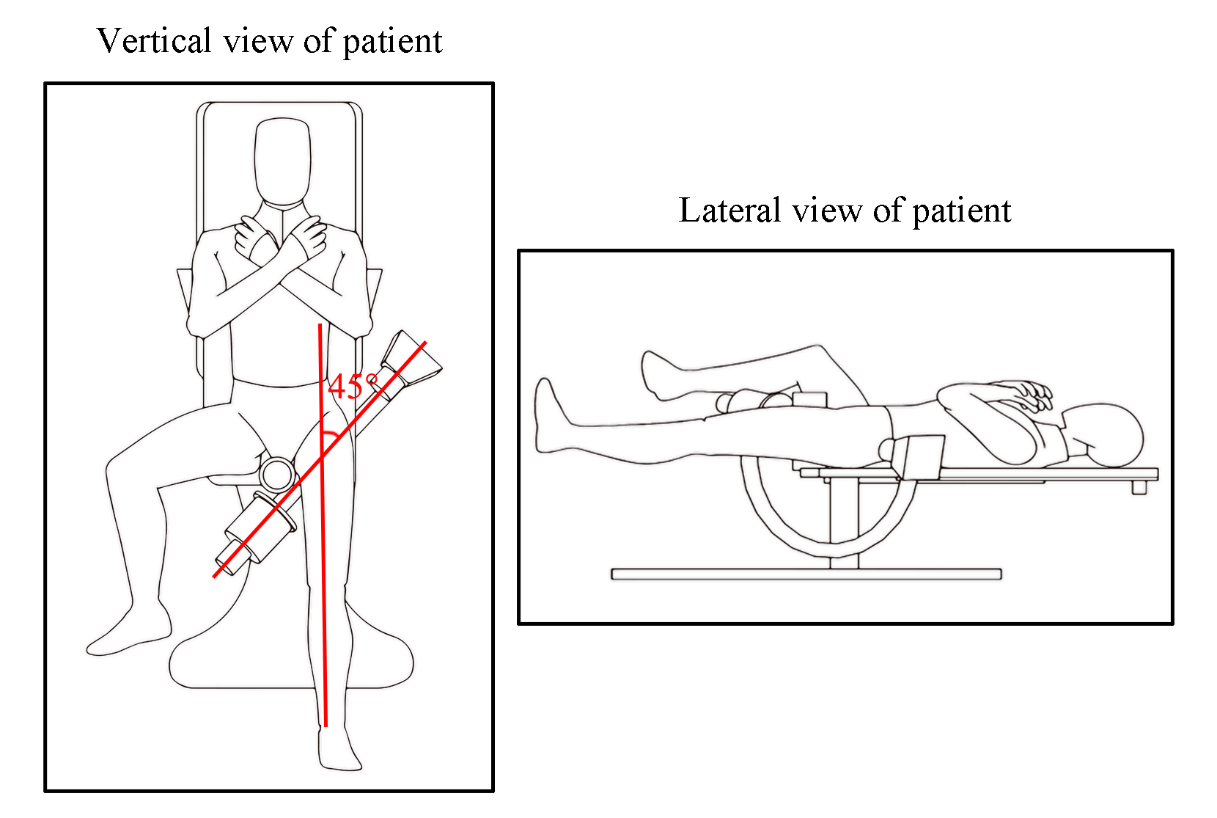
**

Figure S2. The direction of the fluoroscope when obtaining the lateral view of the hip.

**Figure S3**

**
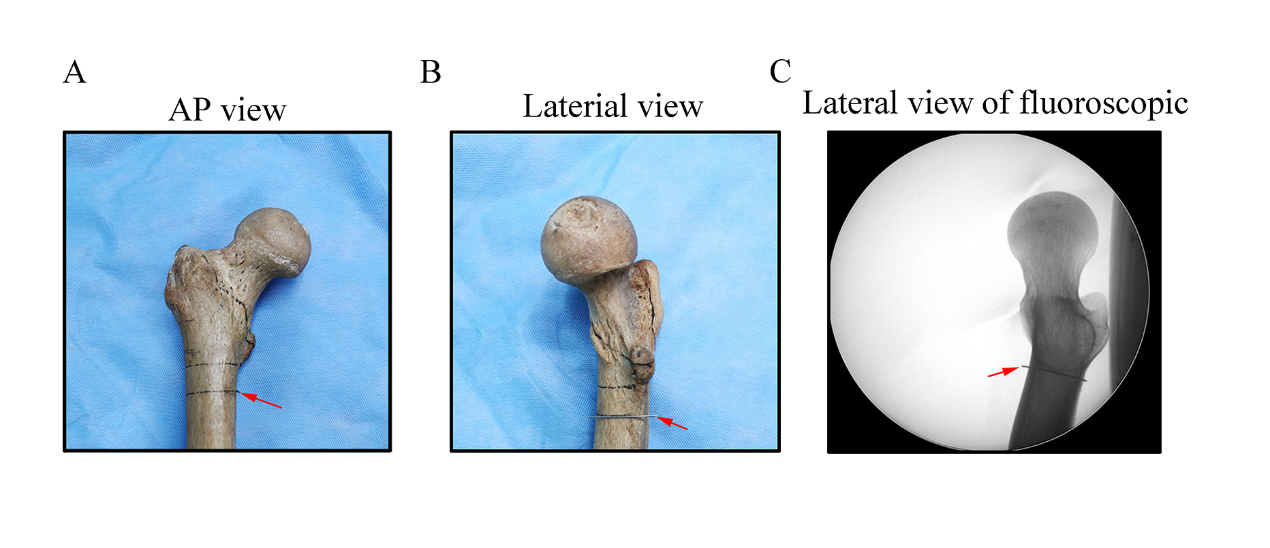
**

Figure S3. Steel wire was employed to mark femoral specimen. A. The femoral specimen was marked by a steel wire in AP view. B. The femoral specimen was marked by a steel wire in the lateral view. C. Steel wire showing the inferior border of the vacuole sign on the lateral radiograph when the direction of the fluoroscopic is 45 degrees to the long axis of femoral specimens on the horizontal plane.

Table S1. The actual position of the inferior border of the vacuole sign on the femoral specimens.

| **Femoral specimen** | **Gender** | **Distance from the lower edge of lesser trochanter (mm)** |
| --- | --- | --- |
| 1 | man | 15.2 |
| 2 | man | 17.3 |
| 3 | man | 14.7 |
| 4 | man | 13.6 |
| 5 | man | 17.2 |
| 6 | women | 14.2 |
| 7 | women | 15.9 |
| 8 | women | 17.2 |
| 9 | women | 15.2 |
| 10 | women | 16.5 |
| **Mean** | / | **15.7±1.26** |
